# Gen-FS coordinated proficiency test data for genomic foodborne pathogen surveillance, 2017 and 2018 exercises

**DOI:** 10.1038/s41597-020-00740-7

**Published:** 2020-11-19

**Authors:** Ruth E. Timme, Patricia C. Lafon, Maria Balkey, Jennifer K. Adams, Darlene Wagner, Heather Carleton, Errol Strain, Maria Hoffmann, Ashley Sabol, Hugh Rand, Rebecca Lindsey, Deborah Sheehan, Joseph D. Baugher, Eija Trees

**Affiliations:** 1 US Food and Drug Administration, College Park, MD USA; 2 Centers for Disease Control and Prevention, Atlanta, GA USA; 3 Association of Public Health Laboratories, Silver Spring, MD USA; 4 International Health Resource Co., Atlanta, GA USA

**Keywords:** Bacterial infection, Pathogens

## Abstract

The US PulseNet and GenomeTrakr laboratory networks work together within the Genomics for Food Safety (Gen-FS) consortium to collect and analyze genomic data for foodborne pathogen surveillance (species include *Salmonella enterica, Listeria monocytogenes, Escherichia coli* (STECs), and *Campylobactor*). In 2017 these two laboratory networks started harmonizing their respective proficiency test exercises, agreeing on distributing a single strain-set and following the same standard operating procedure (SOP) for genomic data collection, running a jointly coordinated annual proficiency test exercise. In this data release we are publishing the reference genomes and raw data submissions for the 2017 and 2018 proficiency test exercises.

## Abstract (summary)

**Table:** 

Measurement(s)	DNA • genome • sequence_assembly
Technology Type(s)	DNA sequencing • sequence assembly process
Factor Type(s)	species of foodborne pathogen
Sample Characteristic - Organism	Salmonella enterica • Escherichia coli • Listeria monocytogenes

Machine-accessible metadata file describing the reported data: 10.6084/m9.figshare.13135046

## Background & Summary

Genomic surveillance of foodborne pathogens in the United States (US) is coordinated by two networks: US PulseNet^1,2^ and GenomeTrakr^3^. PulseNet includes certified public health labs in all 50 US states; its mission is to collect and sequence isolates from human foodborne illnesses and identify outbreaks. The GenomeTrakr network consists of academic, agricultural, international, private, and public health labs; its mission is to sequence the isolates found in food and environmental sources (facility inspections, animals, water, etc), which provides leads for identifying sources of foodborne contamination events. Both networks submit their raw sequence data to the Sequence Read Archive (SRA) database at the National Center of Biotechnology Information (NCBI). These data^4,5^ are also made available within NCBI’s Pathogen Detection portal, which provides updated clustering of related isolates and information about antimicrobial resistance. The procedures for collecting and analyzing these whole genome sequencing (WGS) data are strictly governed by a multi-agency consortium called Genomics for Food Safety (Gen-FS)^6,7^. Members include the Centers of Disease Control and Prevention (CDC), the Food and Drug Administration (FDA), the NCBI, the United States Department of Agriculture (USDA) Food Safety Inspection Service (FSIS), Association of Public Health Laboratories (APHL), and state public health labs. Within Gen-FS, working groups harmonize lab protocols, standardize data quality requirements, verify new technologies, and coordinate an annual proficiency test (PT), among other activities. The PT exercises for PulseNet and GenomeTrakr were held separately until 2017, when the two networks agreed to hold a single annual PT exercise spanning both networks, harmonizing the strain-set and general SOP for data collection.

Each lab participating in PulseNet or GenomeTrakr networks must pass the annual PT to ensure proficiency in collecting WGS data for foodborne pathogens. In addition, these PT exercises are crucial for Gen-FS in accessing overall quality assurance (QA) and quality control (QC) for public health data collection. This exercise helps the Gen-FS coordinating team identify areas for improvement in sequence quality, data transfer, adherence to the SOP, QC thesholds, and communications. For PulseNet participating laboratories, passing the annual PT is a requirement for maintaining the laboratory’s formal certification status which allows the laboratory to submit data to PulseNet’s databases. If the initial PT submission fails, a laboratory is allowed to resubmit twice before losing its certification. For GenomeTrakr participating laboratories, a successful PT submission qualifies as a “pass” (see Technical Verification for detail on QC thresholds used for inclusion) and resulting reports provide a rigerious statistical accessment of the submission. Previously published PT exercises^8,9^ for WGS in foodborne pathogens were able to identify several interesting patterns including a low-rate of genetic variation among clonal isolates and stayed within expected error rates for several key areas, such as sequence quality, read mapping, assembly, insert sizes, and variant detection. Data also showed remarkable uniformity across dozens of laboratories.

Here we present the raw PT data collected from the Gen-FS PT exercises held in 2017 and 2018. For each year’s PT exercise each participating laboratory received the same set of six isolates to sequence according to the Gen-FS harmonized SOP (the isolate sets were different each year). After following the data collection protocol, the labs submitted raw WGS data for each of the six isolates to their respective coordinating team for a full PT analysis (PulseNet, GenomeTrakr, or both). Coordinating teams then reported back to each participating laboratory with a “pass/fail” and an analysis report of the submitted data. The 12 laboratories that were members of both GenomeTrakr and PulseNet received two independently-generated reports back from each respective coordinating team.

For past technologies (e.g. PFGE), the general public or industry stakeholders would have had to issue a Freedom of Information Act (FOIA) request to access data collected from this type of exercise. However, in following our open data commitment across our foodborne pathogen surveillance effort, we are also releasing the data for our PT exercises. The raw sequence data collected, along with closed reference genomes for each of the isolates (6 for each year; total 12) were made public at NCBI.

As new chemistries are adopted within our established WGS workflow and new laboratories join the network, annual PTs play an important role for monitoring consistency while highlighting potential areas of improvement. Combing through these datasets each year enables us to understand laboratory-to-laboratory variation, to provide checks for our QA/QC thresholds, and to ensure proper verification for new chemistries, protocol changes, and new next generation sequencing (NGS) technologies as they come online. These quality assurance steps are important for public health and disease surveillance, and they also provide important transparency for the industries that are most effected by regulatory action (recalls, seizures, injunctions, etc.).

## Methods

### Reference genomes

A different set of strains was chosen for each PT exercise: four *Salmonella enterica* and two Shiga toxin producing *Escherichia coli* (STEC) strains were selected in 2017, and in 2018 four *S. enterica* and two *Listeria monocytogenes* strains were selected. Each of these 12 strains were closed on the Pacific Biosciences (PacBio) *RS II* sequencing platform to provide a baseline to which the results of the participating labs would be compared. The preparation, sequencing of the 20 kb libraries and the subsequent sequence analyses were carried out as described in Timme, R. E. *et al*.^8^. In summary, the libraries were prepared based on the 20 kb PacBio sample preparation protocol. Afterwards the libraries were sequenced using the P6/C4 chemistry on two to three single-molecule real-time (SMRT) cells (with size selection and without) with a 240-min collection time on the Pacific Biosciences *RS II* platform. Analysis of the continuous long reads was implemented using SMRT Analysis 2.3.0. and *de novo* assembly was performed using PacBio hierarchical genome assembly process HGAP 3.0^10^ with default parameters. Resulting assemblies for both chromosomes and plasmids were checked manually for even sequencing coverage and were processed using Gepard^11^ to identify overlapping regions at the ends. The improved consensus sequence was uploaded in SMRT Analysis 2.3.0 to determine the final consensus and accuracy scores using the Quiver consensus algorithm. Further, potential SNPs/indels were corrected with Pilon v1.18^12^ using paired-end short-read data obtained from the Illumina MiSeq platform then mapped to the reference sequences via Bowtie2 v2.2.9^13^.

### Strain distribution

For each PT exercise, participating laboratories each recieved six lyophilized strains. In 2017, 64 laboratories participated; 12 of these labs were participants of both GenomeTrakr and PulseNet. In 2018, 78 laboratories participated, 13 of these labs were participants of both GenomeTrakr and PulseNet. (Online-only Table 1).

### Strain revival for *S. enterica*, *E. coli* and *L. monocytogenes*

The lyophilized cells were resuspended with 1.0 mL sterile reagent grade water or trypticase soy broth (TSB), small amount was inoculated on a blood agar plate (BAP), and incubated overnight at 37 °C. A single, isolated colony was picked and streaked on fresh BAP and incubated at 37 °C overnight in aerobic conditions. The growth from this plate was used to make DNA templates. If no growth occurred on the initial plate, a second attempt was made by re-plating a larger volume of the resuspension.

### DNA extraction, library prep and sequencing

Participating laboratories were instructed to use the Gen-FS harmonized SOP for DNA extraction, library preparation and DNA sequencing. DNA was extracted using Qiagen DNeasy (Qiagen, Hilden, Germany) kits. The libraries were prepared using the Nextera XT (Illumina, San Diego, CA) DNA library prep kit with one of two options: (a) the standard Illumina bead-based normalization or (b) manual normalization using library concentrations and estimated genome size. Sequencing was preformed using MiSeq Reagent Kit v2 (Illumina, San Diego, CA) chemistry for 2 × 250 cycles. Each PT run contained exactly 16 isolates: the 6 PT isolates along with 10 additional routine and/or historical isolates (replicates of the PT isolates were allowed). Participants populated the sequencing sample sheets with the following IDs: “Sample_ID” included the PulseNet proficiency identifiers and technician’s initials; “Sample_name” included the sample ID, Lab ID, machine ID and run date (e.g. SAP18-8999jk-GA-M0947-180215.), and “Project”, which was only used in the GenomeTrakr workflow to create unique folders within BaseSpace for data transfer.

### Data transfer

While the strains and data collection were harmonized across this exercise, the data transfer, analysis and reporting were performed separately within PulseNet and GenomeTrakr for their own member laboratories. Depending on the type of laboratory and their access to data transfer services, there were several possible routes for transferring data. Laboratories with network access to BaseSpace Sequence Hub (Illumina) streamed their sequencing run(s) directly to BaseSpace, then shared their data with their respective network coordinating team(s). Non-federal laboratories without BaseSpace access transferred their raw data (as FASTQ files) through a secure file transfer protocol (SFTP) site. Laboratories within the federal network transferred their runs to an accessible shared drive. Along with the FASTQ files, each laboratory specified which variation in the library prep they followed: (a) the standard Illumina bead-based normalization or (b) manual normalization using library concentrations and estimated genome size.

## Data Records

A single umbrella bioproject, PRJNA504454, and two data bioprojects for 2017^14^ and 2018^15^ were established at NCBI to hold all the data associated with this exercise. Each data BioProject contains six biosamples, describing the metadata for each of the six strains distributed during the respective PT exercises (Online-only Table 2). Complete reference genomes for each distributed strain (annotated, closed assemblies) were submitted to NCBI’s Genbank (Online-only Table 2).The FASTQ files (raw sequence data) from each participating laboratory were submitted to NCBI’s sequence read archive (SRA) database and linked to the appropriate biosample and bioproject (Supplemental File 1). In observing the norm of publishing proficiency test results, we have de-identifed the laboratories from their respective data submissions^16–21^. The individual PT evaluation results for each laboratory are confidential. However, we are extending beyond the norm by releasing all the names of participating laboratories (Online Table 1) *and* the raw data collected across the exercise. PT exercise results are a snapshot in time that may or may not reflect deeper quality control issues in a laboratory. Coordinating bodies worked directly with laboratories that underperformed, identifying and solving any QC issues that appeared systemic. Our goal with this data release is to communicate the value of the entire dataset without fears of public or legal retribution for the participants.

## Technical Verification

### Internal QC to determine validity of data for both 2017 and 2018

In order to ensure maximal utility, the dataset was restricted to samples that passed a series of QC thresholds set by each network. Although the isolates and sequencing protocols were harmonized across the PT exercises, each coordinating body ran their own analyses and distributed their own reports, reflecting each bodies different goals for the exercise (e.g. PulseNet used graded reports because many PN labs need them for accreditation purposes (e.g. CLIA) and GenomeTrakr used a statistical assessment-style report with the goal of using the assessments to identify problem areas and improve overall quality).

**PulseNet** utilized standardized organism-specific evaluation forms and a grading system to evaluate the PT submissions for critical and non-critical quality metrics (Supplemetal Files 2 and 4). Failing to meet the minimum threshold/acceptable range for any of the critical quality metrics resulted in an automatic failure, while failing to meet the minimum threshold for the non-critical metric (insert size) resulted in points deduction. A maximum of 100 points could be accumulated and a minimum of 85 points was required for passing. The following rejection criteria were used for the critical quality metrics:

1. Average coverage < 20x for *Listeria*, < 30x for *Salmonella*, and < 40x for *Escherichia*
2. Read 1 and Read 2 average Q score < 28.00. Sequences with quality scores 28.00–29.99 were accepted with 10–20x additional coverage but resulted in points deduction
3. Assembled genome size > 5% outside the expected size
4. Percentage of core genome genes detected < 95% (*Listeria* only)
5. Number of hqSNP differences > 1 per megabase compared to the reference sequence
6. Number of cgMLST allele differences >3 compared to the reference sequence

### Analysis summary for PulseNet

In 2017, of the 31 participating laboratories, 30 passed the PT for *Salmonella* and STEC with an average passing score of 99 for each organism (Supplemental File 2). In 2018, of the 46 participating laboratories, 42 passed for *Listeria* with an average passing score of 96 and 40 passed for *Salmonella* with an average score of 97 (Supplemental File 4). The failed submissions were caused by low average coverage or quality score, incorrect genome size, low percentage of core genome detected and apparent isolate mix-ups as evidenced by high allele and hqSNP differences compared to the reference sequence. Many labs also appeared to struggle in meeting the minimum insert size of 300 bp. No laboratories lost their certification status as resubmissions were successful. As a follow-up to the detected insert size problem, PulseNet developed focused troubleshooting and training materials materials on improving the insert length and recommended that the laboratories switch from the Nextera XT library preparation kit to the newer Nextera DNAFlex kit.

**GenomeTrakr** excluded any individual samples failing to meet the minimal expected thresholds for average coverage (<20X) and average read quality (Q score < 28.00). Entire sequencing runs were excluded (equivilant to a PulseNet failure) for the following reasons:

1. Any evidence of sample misannotation
2. Any evidence of noncompliance with the SOP (including read length, library prep kit, sequencing chemistry, and number of samples per run)
3. Runs which included too few acceptable PT samples (<4).

Submissions that passed this initial screening were included in the exercise and a statistical report was generated placing each PT submission in context with data from the entire exercise (example reports included in Supplemental Files 3 and 5). Labs were given the option to re-sequence the panel if their submission included QC thresholds far outside the normal range (lower and upper quartiles) in an effort to encourage the labs to make improvements within their laboratory workflows based on feedback from their PT assessment.

## Usage Notes

We see many possible uses for this dataset, especially to demonstrate the reliability of sequencing data produced by large networks of diverse laboratories. We encourage the use of this dataset for competency assessments, verifying or validating new chemistries and platforms, and PT:

1. Obtain and sequence, while following the Gen-FS SOP, a subset of the PT isolates described in this manuscript (for access to the strains, please contact PulsenetNGSlab@cdc.gov - the ATCC catalogue numbers for the strains were pending at the time of the publication),
2. Using a bioinformatics analysis pipeline of choice, analyze the new sequencing data along with the relevant subset of data described in this manuscript (SRA run accessions for downloading data listed in Supplemental File 1),
3. For each desired analytical metric, assess the new data as part of a distribution representing all of the available runs of the same isolate.

Importantly, in contrast to single threshold-based assessments, these data enable individual assessments using standard statistical methods such as interquartile range and outlier detection (see example statistical analyses in Supplemental Files 3 and 5). This allows laboratories to gain the feedback of participating in large-scale PT exercise without having to participate directly.

## Supplementary information

Supplementary File 1

Supplementary Files 2-5

## Online-only Tables

**Online-only Table 1:** Participating laboratories in the 2017 and 2018 proficiency tests.

Laboratory	GT / PN participation	2017	2018
NOVA South Eastern University	GT	x	—
JIFSAN, Joint Institute for Food Safety and Applied Nutrition	GT	x	—
FDA/CFSAN Moffett Center	GT	x	—
FDA Winchester Engineering and Analytical Center (WEAC, FMA)	GT	x	—
FDA Forensic Chemistry Center (FCC)	GT	x	—
Brigham Womens Hospital, Lynn Bry’s lab	GT	x	—
West Virginia Department of Health and Human Resources	PN	—	x
State of Maine Health and Environmental Testing Laboratory	PN	—	x
South Dakota State Department of Health	PN	—	x
Public Health Agency of Canada	PN	—	x
Oregon State Public Health Laboratory	PN	—	x
New York City Department of Health	PN	—	x
New Mexico Department of Health	PN	—	x
Nebraska Public Health Laboratory	PN	—	x
Mississippi State Department of Health	PN	—	x
Louisiana Department of Health & Hospitals	PN	—	x
Los Angeles County Public Health Laboratory	PN	—	x
Kentucky Health Services Laboratory	PN	—	x
Center for Disease Control and Prevention	PN	x	—
Institute of Environmental Science & Research Limited Christchurch Science Centre	PN	—	x
Idaho Bureau of Laboratories	PN	—	x
Florida Department of Health	PN	—	x
FDA Center for Veterinary Medicine (CVM)	PN	—	x
City of Houston Department of Health and Human Services	PN	—	x
Arizona State Public Health Laboratory	PN	—	x
Wyoming Public Health Laboratory	PN	x	x
Wisconsin State Laboratory of Hygiene	PN	x	x
Washington State Department of Health	GT/PN	x	x
Virginia Division of Consolidated Laboratories	GT/PN	x	x
Utah Public Health Laboratory	PN	x	x
Texas Department of State Health Services	GT/PN	x	x
Tennessee Department of Health Laboratory Services	PN	x	x
State Hygienic Laboratory University of Iowa	GT/PN	x	x
South Dakota State Department. of Health / SD State University	GT	x	x
SENASICA - Sequencing and Bioinformatic Unit	GT	x	x
Santa Clara County Public Health Laboratory	PN	x	x
Rhode Island Department of Health	PN	x	x
Pennsylvania State University, Dudly Lab	GT	x	x
Orange County HCA Public Health Laboratory	PN	x	x
Oklahoma State Department of Health	PN	x	x
Ohio Department of Health Laboratory	PN	x	x
Ohio Department of Agriculture, Animal Diseases Diagnostic Lab.	GT	x	x
NSF International | Applied Research Center	GT	x	x
North Dakota Public Health Laboratory	PN	x	x
North Carolina State University, College of Veterinary Medicine	GT	x	x
North Carolina State Laboratory of Public Health	PN	x	x
New York State Department of Health - Wadsworth	GT/PN	x	x
New York State Department of Agriculture & Markets	GT	x	x
New Mexico State University, Food Safety Laboratory	GT	x	x
New Hampshire State Public Health Laboratory	PN	x	x
Nestle Ltd - Nestle Research Center	GT	x	x
Montana Public Health Laboratory	PN	x	x
Minnisotta Department of Health	GT/PN	x	x
Michigan Department of Community Health	PN	x	x
Michigan Department of Agriculture and Rural Development	GT	x	x
Massachusetts Department of Public Health	GT/PN	x	x
Maryland Department of Health and Mental Hygiene	GT/PN	x	x
Lousiana LA-Vet-LIRN	GT	x	x
Indianna State Department of Health Laboratory	PN	x	x
IFSH HTS Laboratory, Moffett Center	GT	x	x
IEH Laboratories & Consulting Group	GT	x	x
Hawaii Department of Health	GT/PN	x	x
Georgia Department of Community Health	PN	x	x
FSIS Office of Public Health Science Athens Field Service Lab	GT/PN	x	x
Florida Dept. of Agriculture and Consumer Services	GT	x	x
FDA CFSAN Office of Applied Research and Safety Assessment	GT	x	x
FDA CFSAN Office of Regulatory Science	GT	x	x
FDA Southeast Food and Feed Laboratory (SFFL)	GT	x	x
FDA San Francisco Laboratory (SANFL)	GT	x	x
FDA Pacific Southwest Food and Feed Laboratory (PSFFL)	GT	x	x
FDA Pacific Northwest Laboratory (PNL)	GT	x	x
FDA Northeast Food and Feed Laboratory (NRL, FNE)	GT	x	x
FDA Gulf Coast Seafood Laboratory	GT	x	x
FDA Denver Laboratory (DENL)	GT	x	x
FDA Arkansas Laboratory (ARKL)	GT	x	x
Delaware Public Health Laboratory	PN	x	x
Cornell University, Vet-LIRN	GT	x	x
Connecticut State Public Health Laboratory	PN	x	x
Colorado Department of Public Health and Environment	PN	x	x
California Department of Public Health, Food and Drug Laboratory	GT/PN	x	x
California Department of Public Health	PN	x	x
Arizona State Public Health Laboratory, Tgen	GT	x	x
Argentina, INEI ANLIS Carlos Malbran Institute	GT	x	x
Alaska Public Health Laboratory	GT/PN	x	x

GT: GenomeTrakr, PN: PulseNet, x: participated in that year’s exercise.

**Online-only Table 2:** Bacterial strains distributed in the 2017 and 2018 proficiency tests exercises.

				Reference genomes and Raw Data colllected from PT exercise		
Year	Organism	Strain	Plasmids	Assembly	NCBI Biosample	NCBI BioProject
2017	*Salmonella enterica* subsp. *enterica* serovar Typhimurium	SAP17-7399	1	GCA_005885855.1	SAMN11787767	PRJNA507262
2017	*Salmonella enterica* subsp. *enterica* serovar Typhimurium	SAP17-7699	1	GCA_005885875.1	SAMN11787765	PRJNA507262
2017	*Salmonella enterica* subsp. *enterica* serovar Typhimurium	SAP17-7299	1	GCA_005885895.1	SAMN11787766	PRJNA507262
2017	*Salmonella enterica* subsp. *enterica* serovar Typhimurium	SAP17-8290	1	GCA_005885935.1	SAMN11786967	PRJNA507262
2017	*Escherichia coli* O157:H7	ECP17-1298	1	GCA_005885915.1	SAMN11787763	PRJNA507262
2017	*Escherichia coli* O157:H7	ECP17-46	1	GCA_005885955.1	SAMN11786966	PRJNA507262
2018	*Salmonella enterica* subsp. *enterica* serovar Typhimurium	SAP18-6199	1	GCA_006351805.1	SAMN09943633	PRJNA507264
2018	*Salmonella enterica* subsp. *enterica* serovar Enteritidis	SAP18-0432	1	GCA_005889955.1	SAMN09943619	PRJNA507264
2018	*Salmonella enterica* subsp. *enterica* serovar Enteritidis	SAP18-H9654	1	GCA_005889975.1	SAMN09943628	PRJNA507264
2018	*Salmonella enterica* subsp. *enterica* serovar Newport	SAP18-8729	2	GCA_004224885.2	SAMN09943626	PRJNA507264
2018	*Listeria monocytogenes*	LMP18-H2446	1	GCA_003900515.2	SAMN09692985	PRJNA507264
2018	*Listeria monocytogenes*	LMP18-H8393	1	GCA_004142705.2	SAMN09692929	PRJNA507264
