# Supplementary material for: Gen-FS coordinated proficiency test data for genomic foodborne pathogen surveillance, 2017 and 2018 exercises
Source: Sci Data. 2020 Nov 19;7:402. doi: 10.1038/s41597-020-00740-7 (PMC7677400; doi:10.1038/s41597-020-00740-7)
Supplement: Supplementary file 2 — Supplementary Files 2-5 [file 41597_2020_740_MOESM2_ESM.pdf]

Supplementary Files:

Table of Contents:

**Supplementary Files**	**Page #**
Supplementary File 2	2
Supplementary File 3	4
Supplementary File 4	19
Supplementary File 5	23

Supplementary File 2. PulseNet proficiency test results for 2017.

Table S1. 2017 Proficiency testing grade distribution among PulseNet participating laboratories

Points scored	*Salmonella*[1]	STEC[1]
**<85**	1	1
**85-90**	0	0
**91-95**	2	1
**96-100**	28	29

[1] Number of laboratories in each points category

Report 1. Example 2017 PulseNet proficiency test reports for *Salmonella* and STEC, both prepared for anonymized laboratory #84. Names of laboratory and people have been removed from the report.

1st PulseNet WGS Proficiency Testing Report Form (Winter 2017)

Organism: Salmonella enterica

Laboratory: #84 Date strains shipped: 1/30/2017 Date results received: 3/22/2017

	Possible Points	No. of Points Received	Comments
**I. Critical Quality Metrics (70 pts)**			
A. Average coverage (>30x)	Pass/Fail	Pass	SAP17-7299 (86x), SAP17-7399 (116x), SAP17-7699 (73x); SAP17-H8290 (73x)
B. Average insert size (>300)	Pass/Fail	Pass	SAP17-7299 (497 bp), SAP17-7399 (493 bp), SAP17-7699 (509 bp); SAP17-H8290 (511 bp)
C. Assembled genome size (4.4 MB, 5% error margin)	Pass/Fail	Pass	SAP17-7299 (4.9Mbp), SAP17-7399 (4.8Mbp), SAP17-7699 (4.9Mbp); SAP17-H8290 (4.9Mbp)
D. Number of hqSNPs compared to reference (< 1 / MB)	Pass/Fail	Pass	SAP17-7299 (0 SNPs), SAP17-7399 (0 SNPs), SAP17-7699 (0 SNPs); SAP17-H8290 (0 SNPs)
E. Number of allele differences compared to reference (≤ 3)	Pass/Fail	Pass	SAP17-7299 (0 alleles), SAP17-7399 (0 alleles), SAP17-7699 (0 alleles); SAP17-H8290 (0 alleles)
**II. Additional Metrics (30 pts)**			
A. Q-score R1 (>30)	8 points	8	SAP17-7299 (36.6), SAP17-7399 (36.3), SAP17-7699 (36.4); SAP17-H8290 (36.4)
B. Q-score R2 (>30)	8 points	8	SAP17-7299 (35.4), SAP17-7399 (35.2), SAP17-7699 (34.8); SAP17-H8290 (34.5)
C. PT isolates run with routine isolates	4 points	4	
D. Results correctly submitted to BaseSpace or FTP	4 points	4	
E. Fastq files named correctly	4 points	4	
F. Submission email sent to PulseNet@cdc.gov	2 points	2	
Total points received		100	
Overall proficiency testing result	Pass or Fail	Pass	

WGS Performed by:

Results Submitted by:

Equipment used: Illumina MiSeq

Chemistry used: V2 500 cycle kit and Nextera Library Prep

Comments:

All items in section I on the report form are in Pass / Fail format. If you fail any one of these sections, you fail the proficiency testing round. Laboratories that pass will accumulate the specified points. For quality metrics, all files were compared to the PulseNet WGS Quality Metrics (SOP appendix PNQ09-5). A passing score is >=85% (>=85/100). The methods used in the analysis of this sequence data are preliminary and remain under validation.

Proficiency Testing Evaluation:

Performed By: ____________________ ______ **Date of Report:**

Performed By: __________________ _____________ **Date of Report:**

Reviewed By: ____________________ _____________ **Date of Review:**

Approved By: ________________ __________ **Date of Approval:**

1st PulseNet WGS Proficiency Testing Report Form (Winter 2017)

Organism: STEC

Laboratory: #84 | Date strains shipped: 1/30/2017 | Date results received: 3/21/17

	Possible Points	No. of Points Received	Comments
**I. Critical Quality Metrics (70 pts)**			
A. Average coverage (>40x)	Pass/Fail	Pass	ECPT17-46 (60X);<br>ECPT-1298 (60X)
B. Average insert size (>300)	Pass/Fail	Pass	ECPT17-46 (509 bp);<br>ECPT17-1298 (508 bp)
C. Assembled genome size (5.5 MB, 5% error margin)	Pass/Fail	Pass	ECPT17-46 (5.4MB);<br>ECPT-1298 (5.3MB)
D. Number of hqSNPs compared to reference (< 1 / MB)	Pass/Fail	Pass	ECPT17-46 (0 SNPs);<br>ECPT17-1298 (1 SNP)
E. Number of allele differences compared to reference (≤ 6)	Pass/Fail	Pass	ECPT17-46 (0 allele diff.)<br>ECPT17-1298 (3 allele diff.)
**II. Additional Metrics (30 pts)**			
A. Q-score R1 (>30)	8 points	8	ECPT17-46 (35.8);<br>ECPT17-1298 (36.8)
B. Q-score R2 (>30)	8 points	8	ECPT17-46 (30.1);<br>ECPT17-1298 (35.3)
C. PT isolates run with routine isolates	4 points	4	
D. Results correctly submitted to BaseSpace or FTP	4 points	4	
E. Fastq files named correctly	4 points	4	
F. Submission email sent to PulseNet@cdc.gov	2 points	2	
Total points received		100	
Overall proficiency testing result	Pass or Fail	Pass	

WGS Performed by:

Results Submitted by:

Equipment used: Illumina MiSeq instrument ID

Chemistry used: Illumina MiSeq V2 (500 cycle)

Comments: Nice work!

All items in section I on the report form are in Pass / Fail format. If you fail any one of these sections, you fail the proficiency testing round. Laboratories that pass will accumulate the specified points. For quality metrics, all files were compared to the PulseNet WGS Quality Metrics (SOP appendix PNQ09-5). A passing score is >=85% (>=85/100). The methods used in the analysis of this sequence data are preliminary and remain under validation.

Proficiency Testing Evaluation

Performed By: | **Date of Report:**

Performed By: | **Date of Report:**

Reviewed By: | **Date of Review:**

Approved By: | **Date of Approval:**

Supplementary File 3. 2017 GenomeTrakr proficiency test report

Report 2. Example 2017 GenomeTrakr proficiency test report prepared for anonymized laboratory #80, shown against the distribution of runs from all participating GenomeTrakr labs. Names of laboratory and people removed from the report.

GenomeTrakr Statistical Report for the 2017 PulseNet-GT harmonized Proficiency Testing Exercise

Joseph Baugher, Ph.D.

May 16, 2017

Contents

1 Overview **2**

2 Results **3**

2.1 Sample Annotation 3

2.2 Data Plots 4

3 Conclusions **14**

4 Additional Information **15**

1 Overview

This report summarizes the results of the whole genome sequencing portion of the 2017 PulseNet-GT harmonized Proficiency Testing (PT) Exercise. This portion of the exercise was intended to assess the ability of a laboratory to perform cell culturing, DNA isolation, and the generation of high quality sequence data using the Illumina MiSeq/Nextera XT system while following the SOP.

Sequencing runs were analyzed using the CFSAN SNP Pipeline v0.7.0 with closed reference sequences generated by the Pacific Biosciences (PacBio) sequencer. Standard metrics are provided by the CFSAN SNP Pipeline. Sequencing run quality was assessed using FastQC v0.11.4. Assembly quality was assessed using the SPAdes Genome Assembler v3.8.0 and QUAST v3.0.

	Isolate ID	Organism
1	SAP17-7299	Salmonella enterica enterica Typhimurium SAP17-7299
2	SAP17-7399	Salmonella enterica enterica Typhimurium SAP17-7399
3	SAP17-7699	Salmonella enterica enterica Typhimurium SAP17-7699
4	SAP17-8290	Salmonella enterica enterica Typhimurium SAP17-8290
5	ECP17-1298	Escherichia coli O157:H7 ECP17-1298
6	ECP17-46	Escherichia coli O157:H7 ECP17-46

Table 1: The 2017 PulseNet-GT harmonized PT strains.

2 Results

2.1 Sample Annotation

No sample annotation problems were detected.

	Sample Name	Expected Isolate	Sequenced Isolate
1	ECP17-1298-M03215	ECP17-1298	ECP17-1298
2	ECP17-46-M03215	ECP17-46	ECP17-46
3	SAP17-7299-M03215	SAP17-7299	SAP17-7299
4	SAP17-7399-M03215	SAP17-7399	SAP17-7399
5	SAP17-7699-M03215	SAP17-7699	SAP17-7699
6	SAP17-8290-M03215	SAP17-8290	SAP17-8290

Table 2: Samples submitted for analysis.

2.2 Data Plots

The following section contains graphical representations of your PT sequencing run (colored data points) and the distribution of runs from all participating GenomeTrakr labs (boxplots with black outlier data points). The isolates are labeled (x -axis) using the numeric portion of the CFSAN ID (see Table 1). For each box plot, the box defines the median value as well as the lower and upper quartiles (25% and 75%). The whiskers extend to the most extreme data point which is no more than 2.5 times the interquartile range from the median.

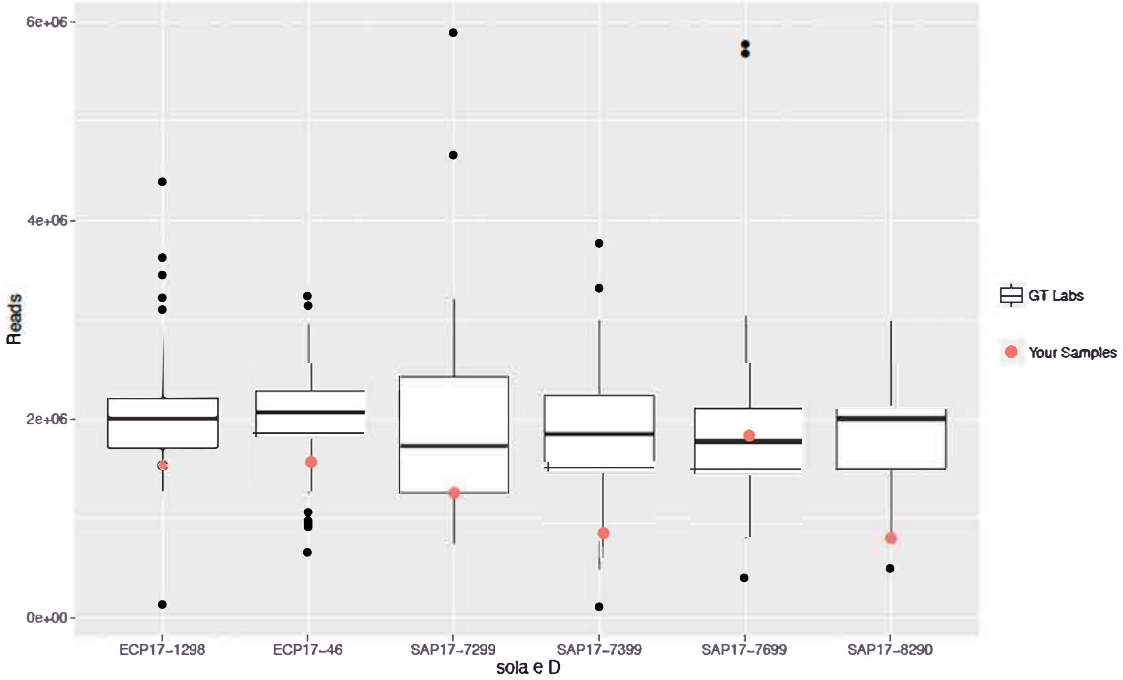

Figure 1: The number of sequencing reads.

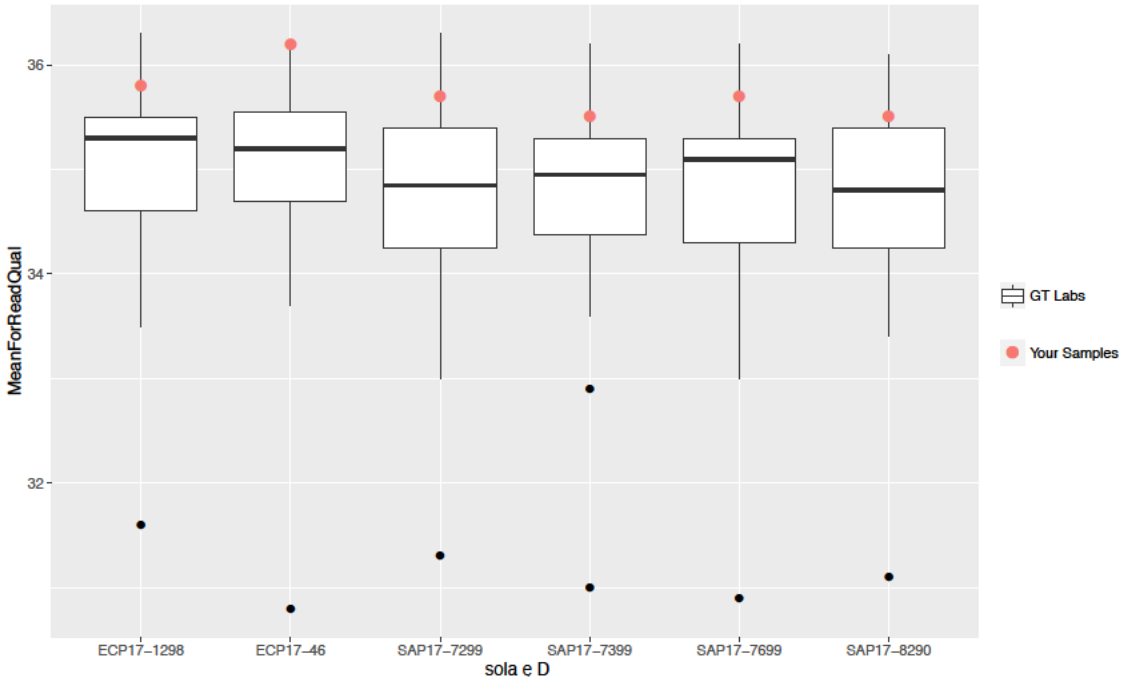

Figure 2: The mean quality score - forward reads.

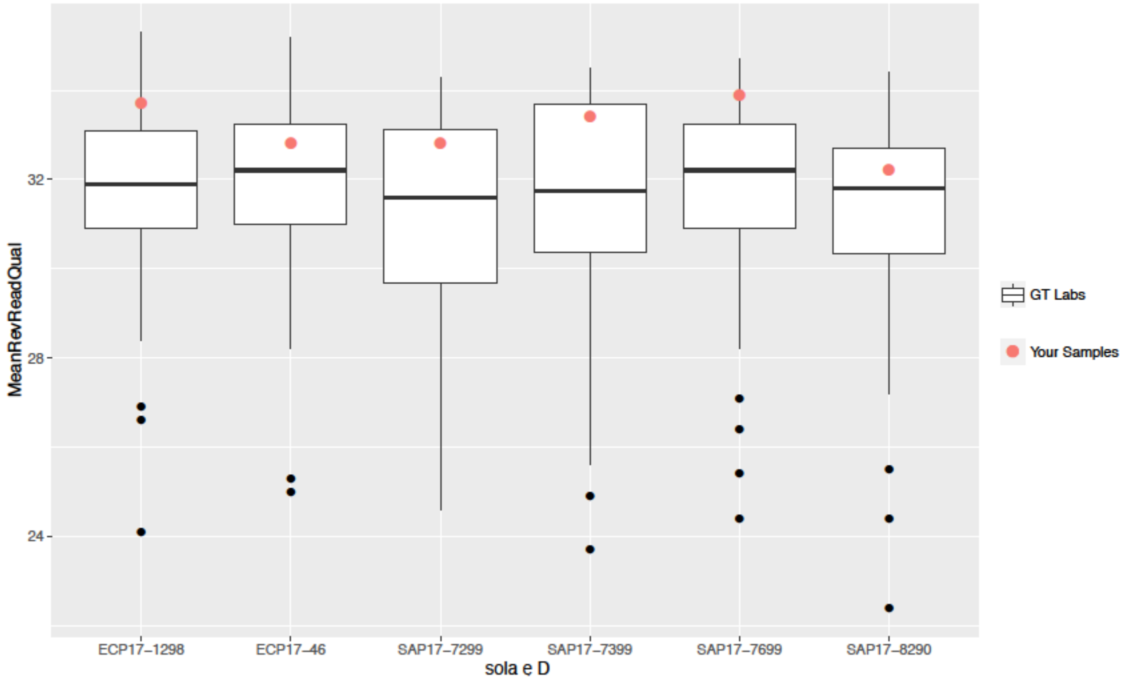

Figure 3: The mean quality score - reverse reads.

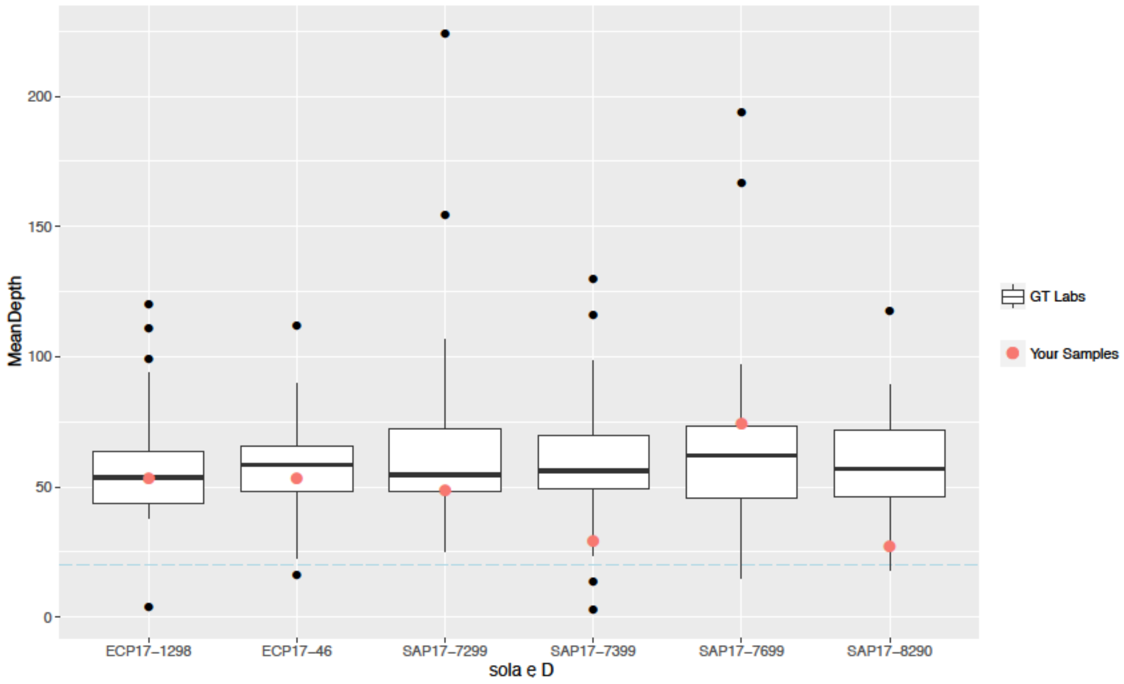

Figure 4: The mean depth of mapped reads. The dashed horizontal line illustrates the current threshold (20X) for sequence submission.

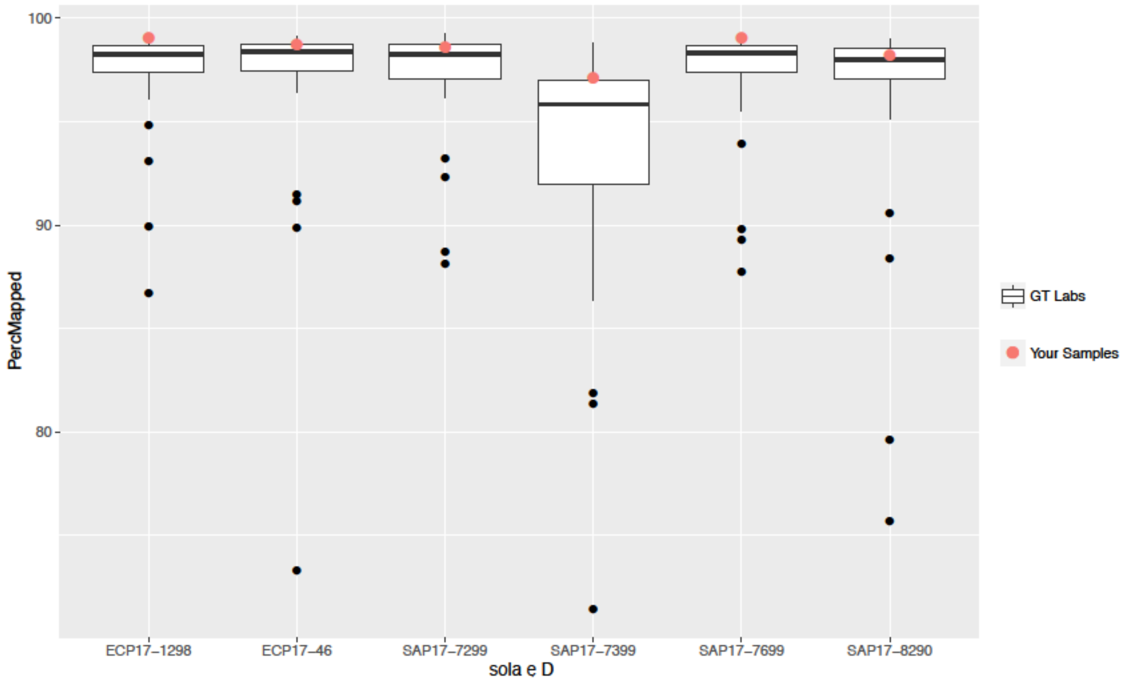

Figure 5: The percentage of reads which could be mapped to the reference genome.

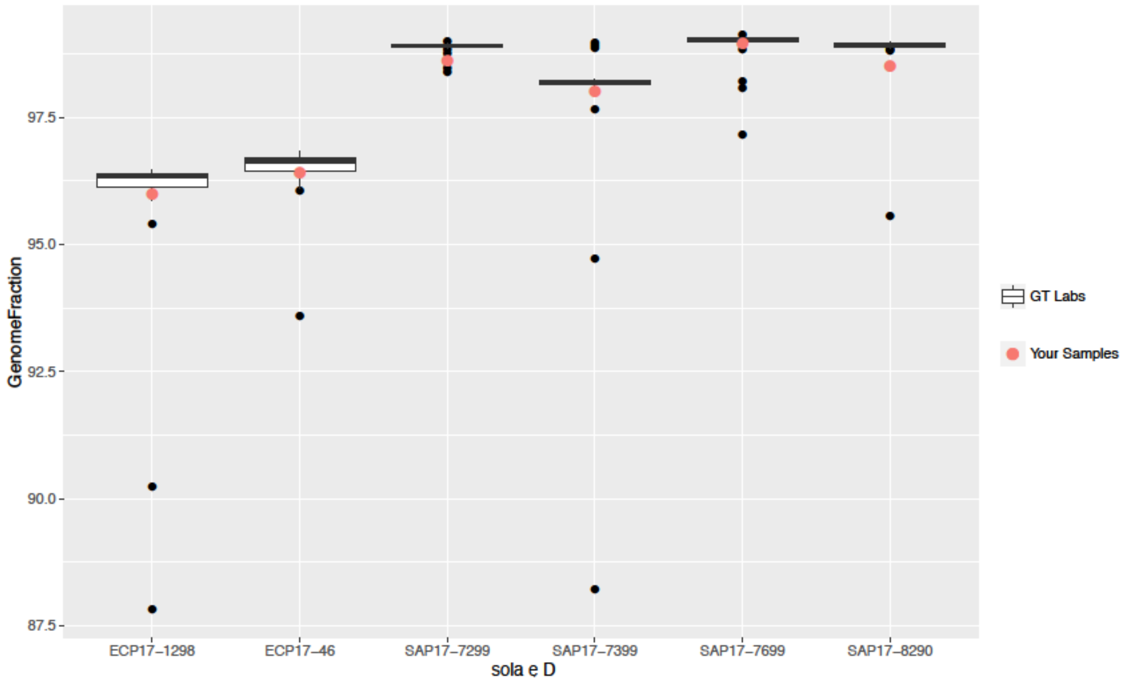

Figure 6: Genome fraction (%). The total number of aligned bases in the reference, divided by the genome size. A base in the reference genome is counted as aligned if at least one contig has at least one alignment to this base. Contigs from repeat regions may map to multiple places, and thus may be counted multiple times in this quantity.

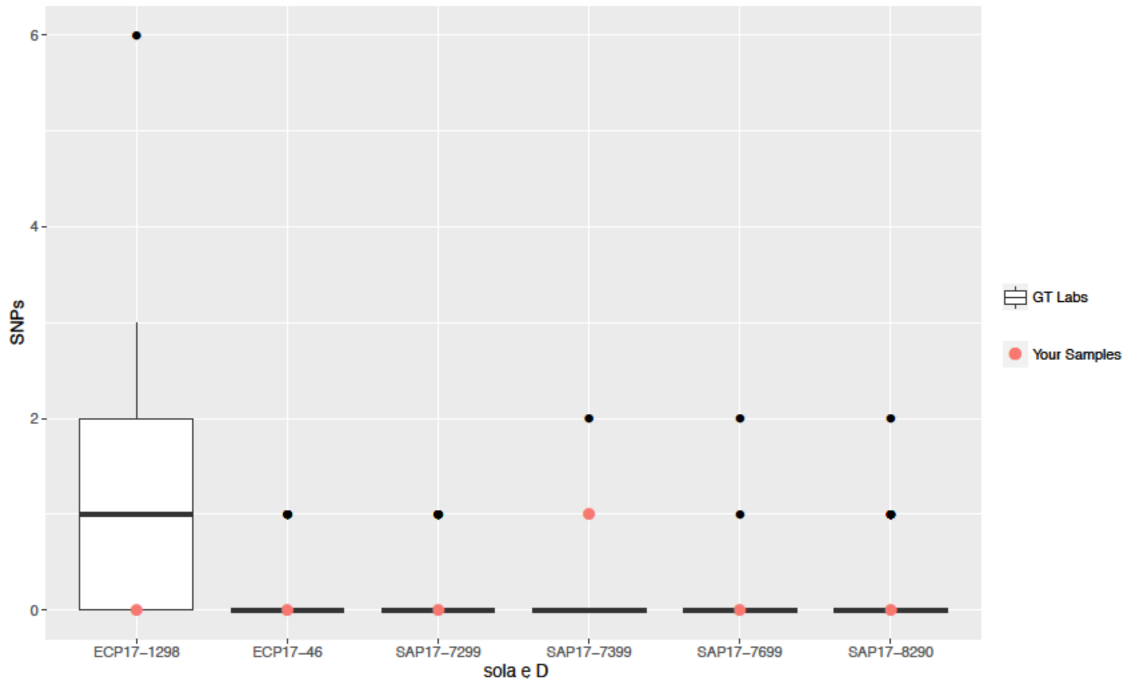

Figure 7: The number of SNPs reported.

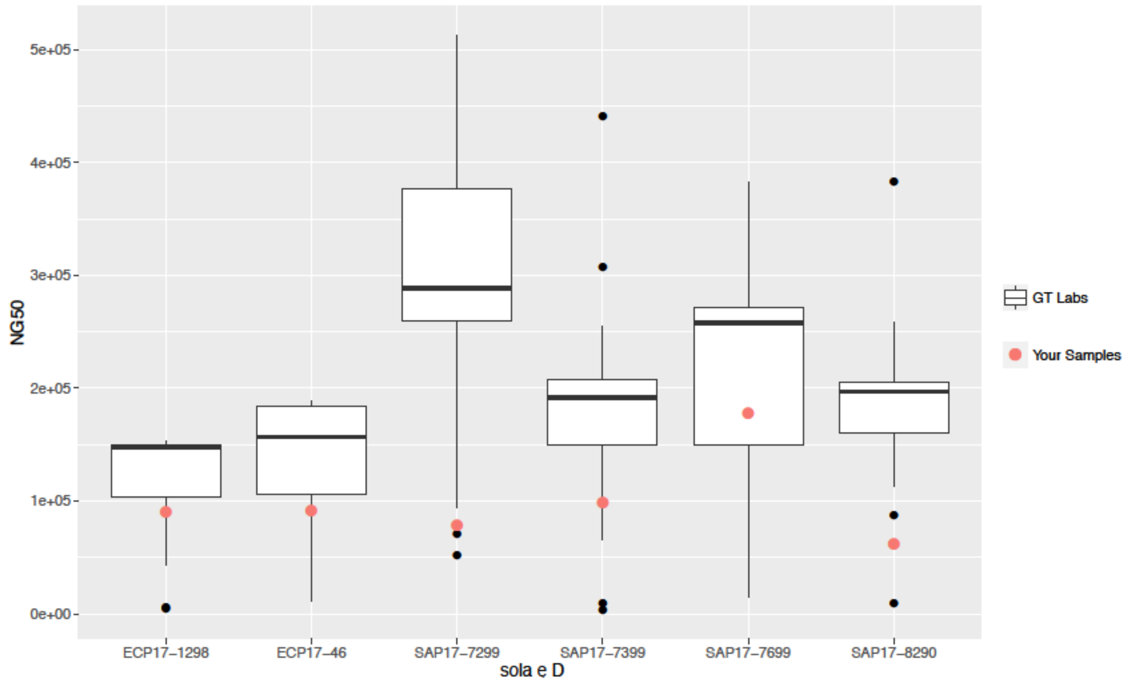

Figure 8: The NG50 assembly quality metric. The contig length such that using equal or longer length contigs produces x% of the length of the reference genome, allowing for comparisons between different genomes. Larger NG50 values generally correlate with a higher quality assembly.

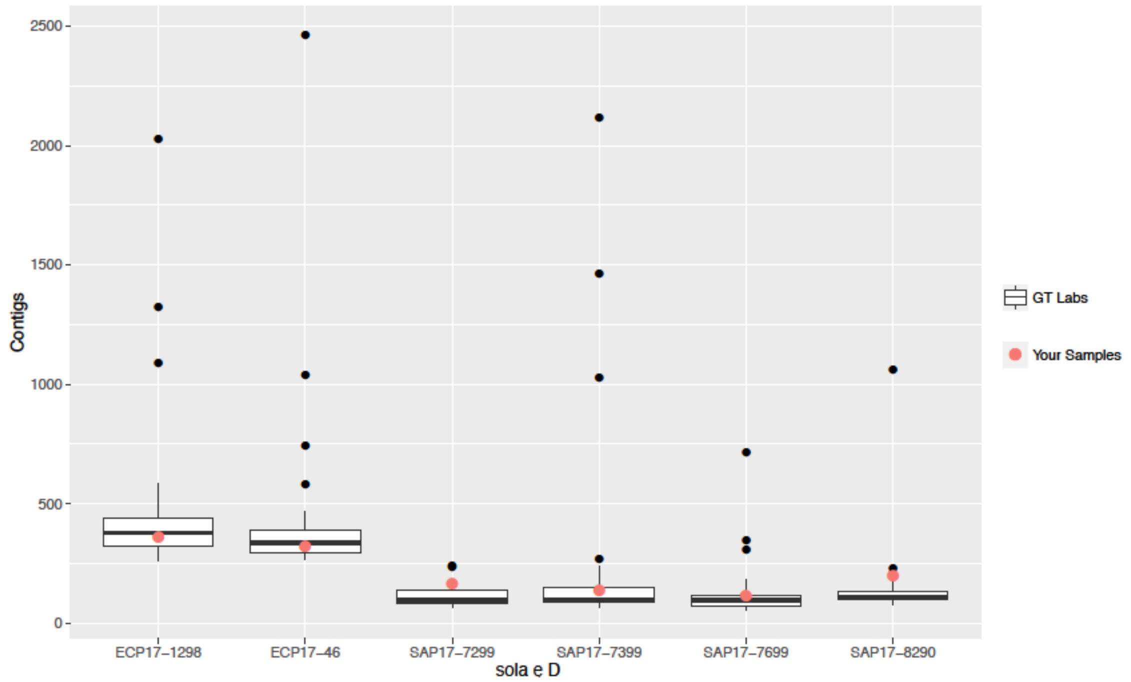

Figure 9: The total number of contigs in the assembly. Fewer contigs generally correlate with a higher quality assembly.

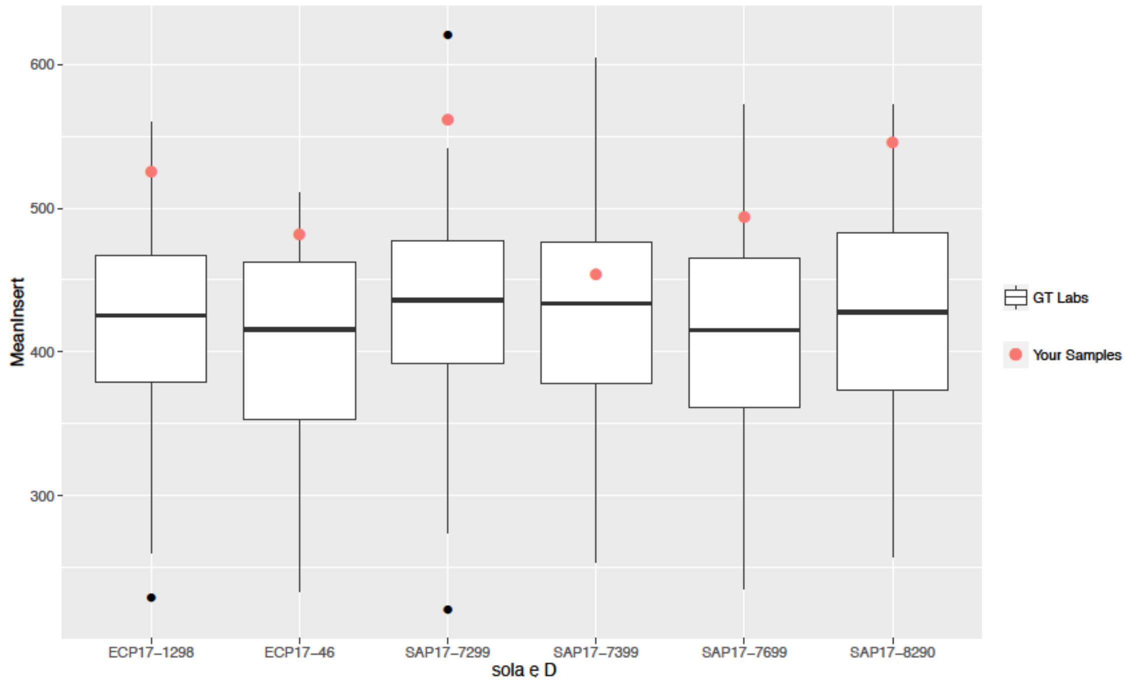

Figure 10: The mean insert size, defined as the length of the sequence between the adapters.

3 Conclusions

The results of the PT analysis revealed that all samples were properly annotated regarding organisms sequenced. Figures 1-10 illustrate the performance of your PT sequencing run using a variety of metrics. Pay particular attention to any figures in which values for your run fall outside of the 25% - 75% interquartile range (the boundaries of the box in the plot).

The mean read depth of sequencing runs is expected to be above the minimum threshold for NCBI submission (20X). Of 6 samples, 6 (100%) were correctly annotated and above the submission threshold.

4 Additional Information

	SampleName	IsolateID	CFSANID	Lab	Sequencer	Machine	FlowCell	LibKit	RunDate	SequencedBy
1	ECP17-1298-	ECP17-1298	CFSAN059541		Illumina MiSeq sequence		000000000-AUTAF	Nextera XT	2017-03-17 22:41:22	
2	ECP17-46-	ECP17-46	CFSAN059540		Illumina MiSeq sequence		000000000-AUTAF	Nextera XT	2017-03-17 22:41:22	
3	SAP17-7299-	SAP17-7299	CFSAN059543		Illumina MiSeq sequence		000000000-AUTAF	Nextera XT	2017-03-17 22:41:22	
4	SAP17-7399-	SAP17-7399	CFSAN059545		Illumina MiSeq sequence		000000000-AUTAF	Nextera XT	2017-03-17 22:41:22	
5	SAP17-7699-	SAP17-7699	CFSAN059544		Illumina MiSeq sequence		000000000-AUTAF	Nextera XT	2017-03-17 22:41:22	
6	SAP17-8290-	SAP17-8290	CFSAN059542		Illumina MiSeq sequence		000000000-AUTAF	Nextera XT	2017-03-17 22:41:22	

Table 3: Run Metadata.

	SampleName	Reads	PercMapped	MeanForReadQual	MeanRevReadQual	MeanDepth	SNPs	MeanInsert	NG50	GenomeFraction	Contigs
1	ECP17-1298-	1539230	99.04	35.80	33.70	53.32	0	524.97	89535	95.97	362
2	ECP17-46-	1616362	98.69	36.20	32.80	53.40	0	481.31	91482	96.42	322
3	SAP17-7299-	1254964	98.60	35.70	32.80	48.43	0	561.73	77996	98.61	165
4	SAP17-7399-	902692	97.10	35.50	33.40	29.04	1	453.88	98206	98.01	134
5	SAP17-7699-	1880370	99.01	35.70	33.90	74.03	0	493.87	177133	98.94	115
6	SAP17-8290-	790590	98.17	35.50	32.20	27.22	0	545.22	62108	98.50	200

Table 4: Run Metadata Continued.

Supplementary File 4. PulseNet proficiency test results for 2018.

Table S2. 2018 Proficiency testing grade distribution among PulseNet participating laboratories

Points scored	*Salmonella*[1]	*Listeria*[1]
**<85**	6	4
**85-90**	3	11
**91-95**	9	7
**96-100**	28	24

[1] Number of laboratories in each points category

Report 3. Example 2018 PulseNet proficiency test reports prepared for anonymized laboratory #59 (*Listeria*) and anonymized laboratory #81 (*Salmonella*). Names of laboratories and people removed from both reports.

2nd PulseNet WGS Proficiency Testing Report Form (Winter 2018)

Organism: *Listeria monocytogenes*

Laboratory: #59 Date strains shipped: 1/22/2018 Date results received: 2/27/2018

I. Sequencing and Fastq file Preparation (80 pts)

	Possible Points	No. of Points Received	Comments
A. Q-score R1 (>30 = 4 pts; 28.00 - 29.99 plus 10-20x additional coverage = 1-3 pts; <28.00 = 0 pts)	Pass (1-4 pts) or Fail (0)	4	LMP18-H8393 (37.16), LMP18-H2446 (36.88)
B. Q-score R2 (>30 = 4 pts; 28.00 - 29.99 plus 10-20x additional coverage = 1-3 pts; <28.00 = 0 pts)	Pass (1-4 pts) or Fail (0)	4	LMP18-H8393 (36.04), LMP18-H2446 (35.74)
C. Average coverage (>20x)	Pass (12 pts) or Fail	12	LMP18-H8393 (203.9X), LMP18-H2446 (130X)
D. Median insert size (>300 = 12 pts; 275 - 299 = 8 pts; <274 = 2 pts)	12 points	7	LMP18-H8393 (227), LMP18-H2446 (433)
E. Assembled genome size (3 MB, 5% error margin)	Pass (12 pts) or Fail	12	LMP18-H8393 (3.0MB), LMP18-H2446 (2.8MB)
F. Percent Core (≥95%)	Pass (12 pts) or Fail	12	LMP18-H8393 (97.8%), LMP18-H2446 (97.7%)
G. Number of hqSNPs compared to reference (< 1 / MB)	Pass (12 pts) or Fail	12	LMP18-H8393 (0), LMP18-H2446 (0)
H. Number of allele differences compared to reference (≤ 3)	Pass (12 pts) or Fail	12	LMP18-H8393 (0), LMP18-H2446 (0)

II. Submission of Results (20 pts)

A. PT isolates sequenced on a full (~80 MB) 500 cycle cartridge	6 points	6	
B. Fastq files named correctly	6 points	6	
C. Results correctly submitted to BaseSpace or FTP	6 points	6	
D. Submission email sent to PulseNet@cdc.gov	2 points	2	

III. Summary of Results

Total points received		95	
Overall proficiency testing result	Pass or Fail	Pass	

WGS Performed by: [redacted]

Results Submitted by: [redacted]

Equipment used: Illumina MiSeq [redacted]

Chemistry used: Illumina MiSeq V2 (500 cycle) and Nextera XT Library Prep Kit

Comments: The median insert size was below 300 bp. Optimizing your library prep with a particular focus on tagmentation and size selection steps will help reach minimum insert size required for certification. Results were confirmed both by mapping back to the reference using SMALT and by generating insert size distribution graphs included below using CLCBio.

LMP18-H2446 Insert Size Histogram:

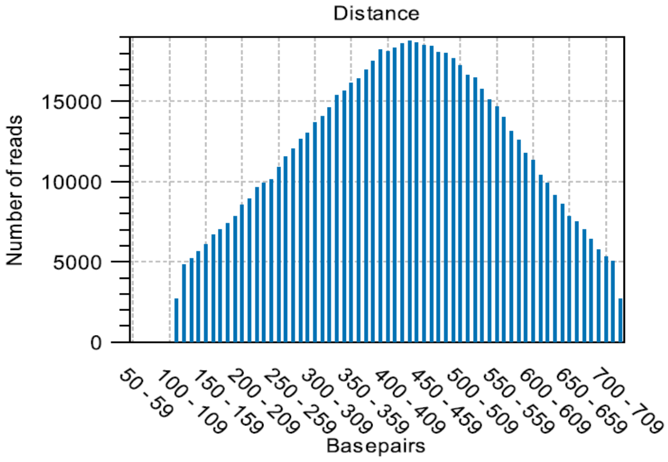

LMP18-H8393 Insert Size Histogram:

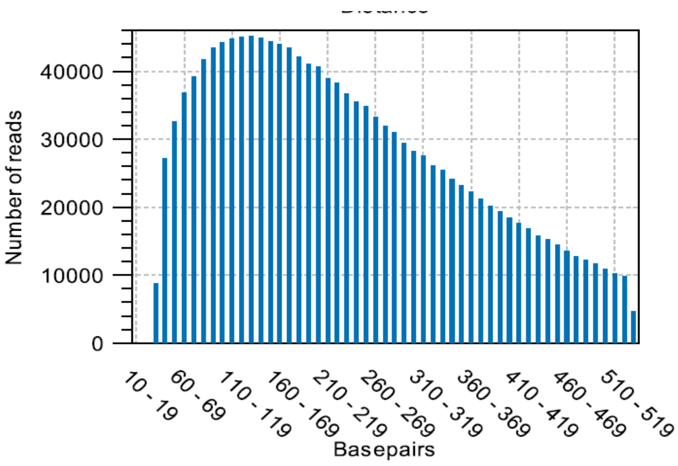

Items in Section I on the report form are in Pass / Fail format. If you fail any one of these sections, you fail the proficiency testing round. Laboratories that pass will accumulate the specified points. For quality metrics, all files were compared to the PulseNet WGS Quality Metrics (SOP appendix PNQ09-5). A passing score is >=85% (>=85/100). The methods used in the analysis of this sequence data are preliminary and remain under validation.

Proficiency Testing Evaluation:

Fastq Evaluation Performed By: **Date:**

Evaluation Performed By: **Date:**

Approved By: **Date:**

2nd PulseNet WGS Proficiency Testing Report Form (Winter 2018)

Organism *Salmonella*

Laboratory: #81 | Date strains shipped: 1/22/2018 | Date results received: 3/19/2018

	Possible Points	No. of Points Received	Comments
**I. Sequencing and Fastq file Preparation (80 pts)**			
A. Q-score R1 (>30 = 4 pts; 28.00 - 29.99 plus 10-20x additional coverage = 1-3 pts; <28.00 = 0 pts)	Pass (1-4 pts) or Fail (0)	4	SAP18-0432 (36.18), SAP18-6199 (36 08), SAP18-8729 (36.2), SAP18-H9654 (36.11)
B. Q-score R2 (>30 = 4 pts; 28.00 - 29.99 plus 10-20x additional coverage = 1-3 pts; <28.00 = 0 pts)	Pass (1-4 pts) or Fail (0)	4	SAP18-0432 (32.71), SAP18-6199 (33.17), SAP18-8729 (33.9), SAP18-H9654 (33.61)
C. Average coverage (>30x)	Pass (15 pts) or Fail	15	SAP18-0432 (133), SAP18-6199 (113 6), SAP18-8729 (164.1), SAP18-H9654 (170.4)
D. Median insert size (>300 = 15 pts; 275 - 299 = 12 pts; <274 = 4 pts)	15 points	15	SAP18-0432 (339), SAP18-6199 (323), SAP18-8729 (302) SAP18-H9654 (327)
E. Assembled genome size (5 MB, 5% error margin)	Pass (14 pts) or Fail	14	SAP18-0432 (4.7), SAP18-6199 (4 9), SAP18-8729 (4 8), SAP18-H9654 (4.7)
F Number of hqSNPs compared to reference (< 1 / MB)	Pass (14 pts) or Fail	Fail	SAP18-0432 (0), **SAP18-6199** **(possible sample swap)**, **SAP18-8729** **(possible sample swap)** SAP18-H9654 (0)
G. Number of allele differences compared to reference (≤ 3)	Pass (14 pts) or Fail	Fail	SAP18-0432 (0), **SAP18-6199** **(possible sample swap)**, **SAP18-8729** **(possible sample swap)**, SAP18-H9654 (1)

II. Submission of Results (20 pts)

A. PT isolates sequenced on a full (~80 MB) 500 cycle cartridge	6 points	6	
B. Fastq files named correctly	6 points	6	
C. Results correctly submitted to BaseSpace or FTP	6 points	6	
D. Submission email sent to PulseNet@cdc gov	2 points	2	

III. Summary of Results

Total points received			
Overall proficiency testing result	Pass or Fail	Fail	

WGS Performed by:

Results Submitted by

Equipment used: Ilumina MiSeq

Chemistry used: Illumina MiSeq V2 (500 cycle) and Nextera XT Library Prep Kit

Comments: It appears there may have been a sample swap with SAP18-6199 and SAP18-8729 based on results for initial hqSNP and wgMLST analysis (number of differences were abnormally high >200 wgMLST and >20,000SNPS). When samples were compared again by swapping these Reference genomes, the hqSNP and wgMLST results were within acceptable limits. SAP18-8729 had 1 hqSNP and 0 allele differences compared to the 6199 Reference Genome. SAP18-6199 had 2 SNP differences and 1 allele difference compared to the 8729 Reference genome.

SAP18-0432 Insert Size Histogram:

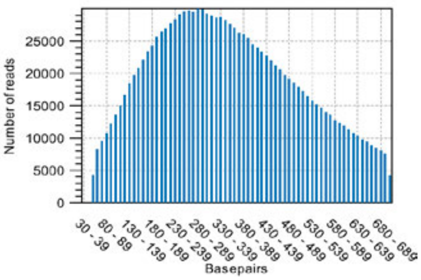

SAP18-6199 Insert Size Histogram:

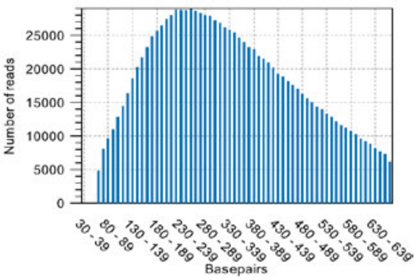

SAP18-8729 Insert Size Histogram:

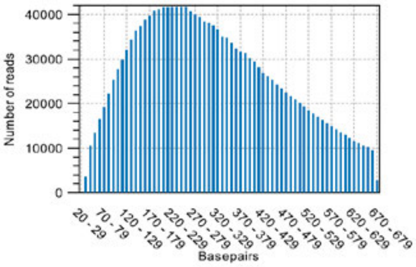

SAP18-H9654 Insert Size Histogram:

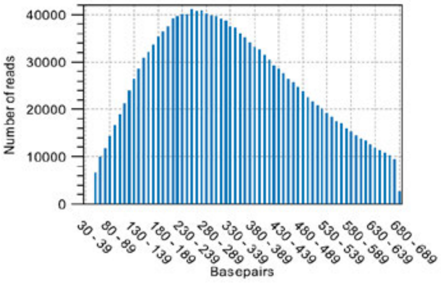

tems in Section I on the report form are in Pass / Fail format. If you fail any one of these sections, you fail the proficiency testing round. Laboratories that pass will accumulate the specified points. For quality metrics, all files were compared to the PulseNet WGS Quality Metrics (SOP appendix PNQ09-5). A passing score is >=85% (>=85/100). The methods used in the analysis of this sequence data are preliminary and remain under validation.

Proficiency Testing Evaluation

Fastq Evaluation Performed By **Date**

Evaluation Performed By **Date**

Approved By **Date**

Report 4. Example 2018 GenomeTrakr proficiency test report prepared for anonymized laboratory #43 shown against the distribution of runs from all participating GenomeTrakr labs. Names of laboratory and people removed from report.

A Statistical Analysis of 2018 Proficiency Testing Isolates

Joseph Baugher, Ph.D.

August 6, 2018

Contents

1 Overview **2**

2 Results **3**

2.1 Run Metrics 3

2.2 Read Metrics 4

2.3 Alignment Metrics 5

2.4 Assembly Metrics 8

3 Conclusions **9**

A Appendix **10**

1 Overview

This report provides graphical visualization of analytical metrics for bacterial WGS data compared to a distribution of isolates sequenced during the 2018 PulseNet-GenomeTrakr harmonized Proficiency Testing exercise as part of a 16-sample 2X250 run on an Illumina MiSeq machine (V2 chemistry) using the Nextera XT DNA Library Prep Kit.

Sequencing runs were analyzed using the CFSAN SNP Pipeline v1.0. Reference sequences were generated by the PacBio SMRT sequencer, closed using the HGAP software, and polished with MiSeq data using pilon v1.22. Standard metrics are provided by the CFSAN SNP Pipeline. Sequencing run quality was assessed using FastQC v0.11.5. Assembly quality was assessed using the SPAdes Genome Assembler v3.11.1 and QUAST v4.5.

	Isolate ID	CFSAN ID	Organism
1	SAP18-0432	CFSAN074386	Salmonella enterica subsp. enterica serovar Enteritidis str. SAP18-0432
2	SAP18-H9654	CFSAN074385	Salmonella enterica subsp. enterica serovar Enteritidis str. SAP18-H9654
3	SAP18-6199	CFSAN074387	Salmonella enterica subsp. enterica serovar Typhimurium str. SAP18-6199
4	SAP18-8729	CFSAN074384	Salmonella enterica subsp. enterica serovar Newport str. SAP18-8729
5	LMP18-H2446	CFSAN074383	Listeria monocytogenes str. LMP18-H2446
6	LMP18-H8393	CFSAN074382	Listeria monocytogenes str. LMP18-H8393

Table 1: The 2018 PulseNet-GT harmonized PT strains.

	Isolate ID	Sample Name	Machine	Flowcell	QC Status
1	H2446	LMP18-H2446-		BDTYL	Passed
2	H8393	LMP18-H8393-		BDTYL	Passed
3	0432	SAP18-0432-		BDTYL	Passed
4	6199	SAP18-6199-		BDTYL	Passed
5	8729	SAP18-8729-		BDTYL	Passed
6	H9654	SAP18-H9654-		BDTYL	Passed

Table 2: PT isolates submitted for analysis.

2 Results

The following section contains graphical representations of your PT sequencing run (red data points) and the distribution of runs from all participating GenomeTrakr labs (boxplots with background data points and black outliers). The isolates are labeled (x -axis) using the Isolate ID (see Table 1). For each box plot, the box defines the median value as well as the lower and upper quartiles (25% and 75%). The whiskers extend to the most extreme data point which is no more than 2.5 times the interquartile range from the median. Note - extreme outliers may have been removed from individual reports in order to improve utility.

2.1 Run Metrics

The following run metrics were evaluated (**Fig. 1**) -

a. **Cluster Density (K/mm^2)** - density of clusters for each tile.
b. **Clusters PF (%)** - the percentage of clusters passing filter for each tile.
c. **Reads PF (M)** - the number of reads/clusters passing filter (millions).
d. **Yield (Gb)** - the total yield in Gigabases.
e. **Bases >= Q30 (%)** - the percentage of bases with a quality score >= 30.
f. **Reads Identified (%)** - the percentage of Reads PF which were assigned to an index.
g. **Indexing CV** - the coefficient of variation for the number of counts across all indices.

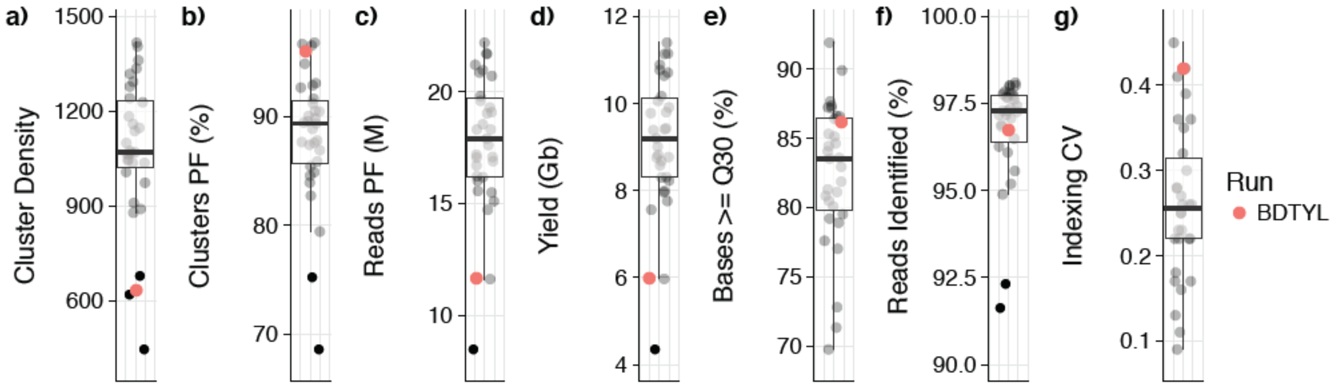

Figure 1: Run Metrics

	BDTYL	Expected
Cluster Density (K/mm2)	633	800-1100
Clusters PF (%)	96.07	
Reads PF (M)	11.66	12-15
Yield (Gb)	5.99	7.5-8.5
Bases >= Q30 (%)	86.21	>75%
Reads Identified (%)	96.75	
Indexing CV	0.42	

Table 3: Run metrics and manufacturer expected values for MiSeq V2 2x250 runs.

2.2 Read Metrics

The following read metrics were evaluated (**Figs. 2-4**) -

2. Reads - the number of read pairs.

3. Mean read quality scores - forward reads (R1) - QC threshold = Q27.

4. Mean read quality scores - reverse reads (R2) - QC threshold = Q27.

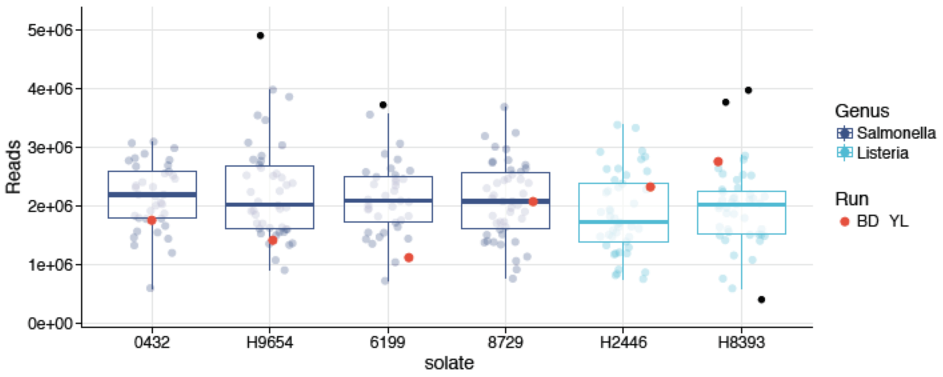

Figure 2: Reads

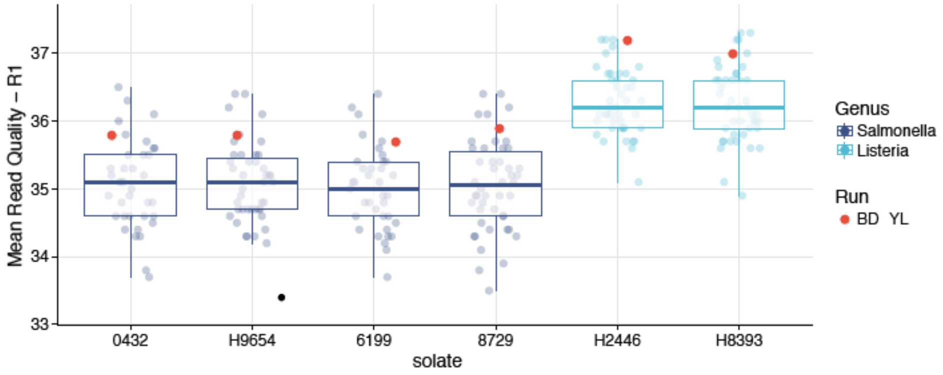

Figure 3: Mean read quality score - forward reads (R1)

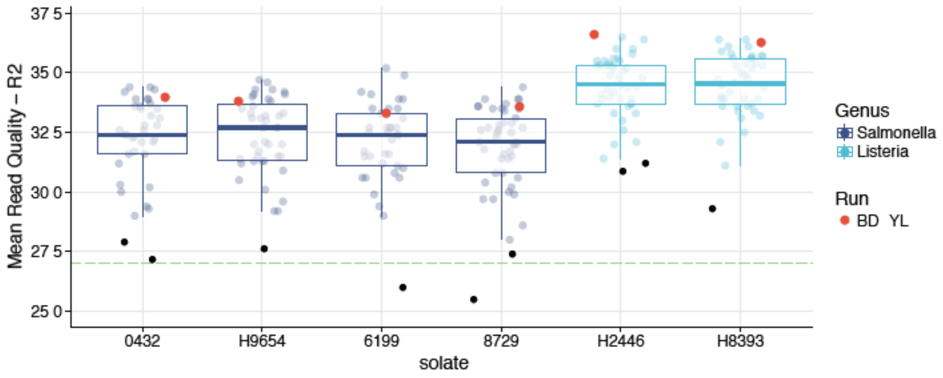

Figure 4: Mean read quality score - reverse reads (R2)

2.3 Alignment Metrics

The following alignment metrics were evaluated (**Figs. 5-11**) -

5. Mean depth - the mean depth of coverage after mapping reads to the reference. QC threshold = 20X.

6. Reads mapped (%) - the percentage of reads which could be mapped to the reference.

7. Genome fraction - the percentage of the reference with >= 1X coverage.

8. Low coverage positions - the number of positions with depth of coverage < 10X. This metric is highly inversely correlated with assembly quality.

9. SNPs - the number of SNPs reported.

10. Mean insert size - the mean length of the sequence between the adapters.

11. Coverage Plots - visualizations of coverage depth and variance across each position. Coverage plots were smoothed to 10000 points. Histograms of the coverage depth should resemble a Poisson-like distribution with a small standard deviation.

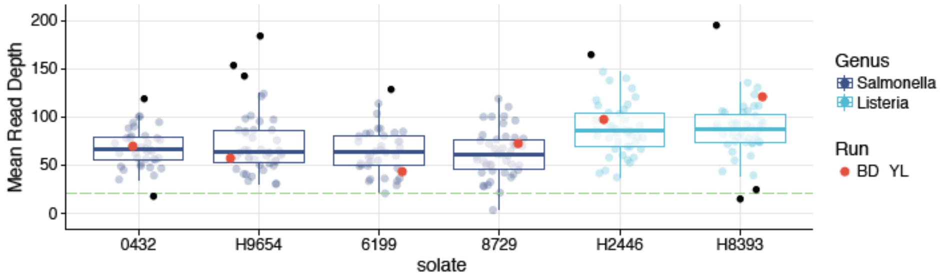

Figure 5: Mean depth

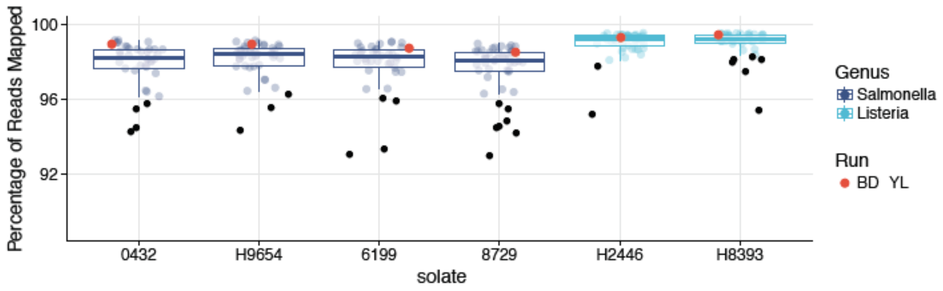

Figure 6: Reads mapped (%)

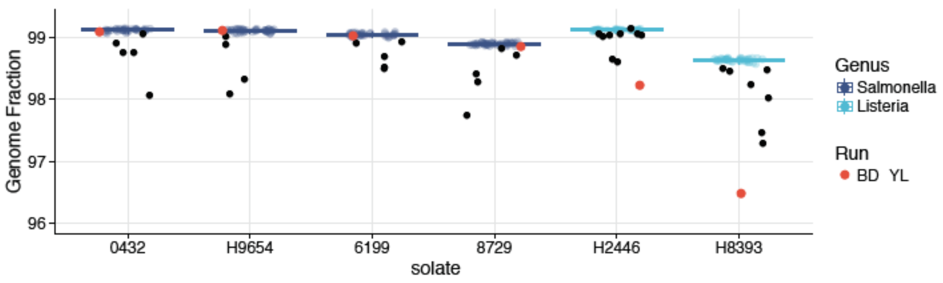

Figure 7: Genome fraction (%)

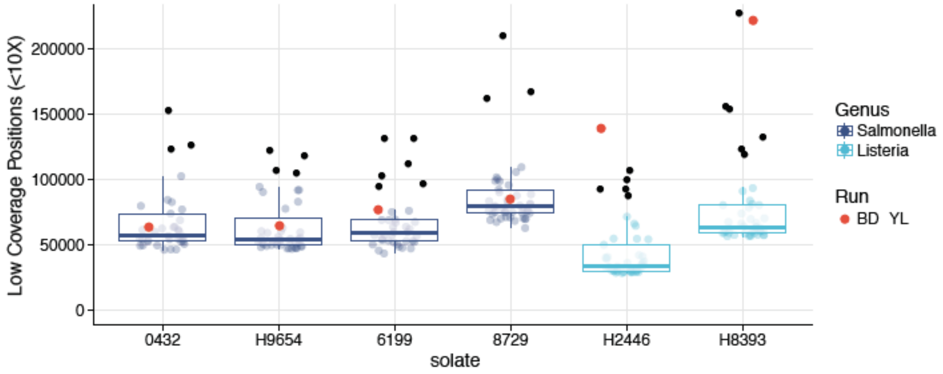

Figure 8: Low coverage positions ($< 10X$)

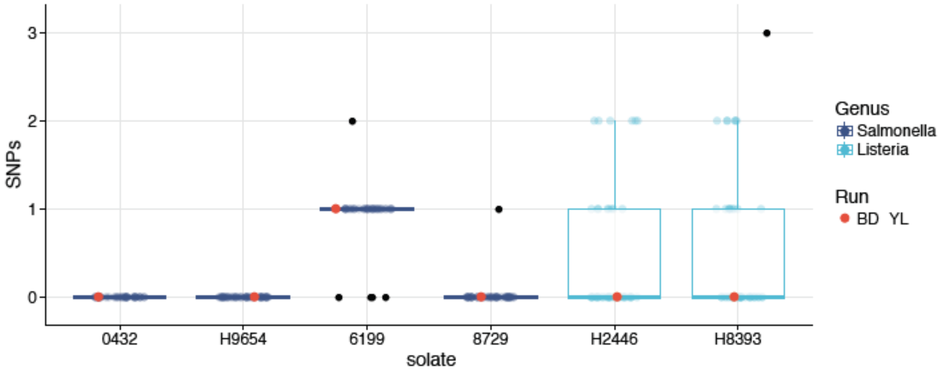

Figure 9: SNPs

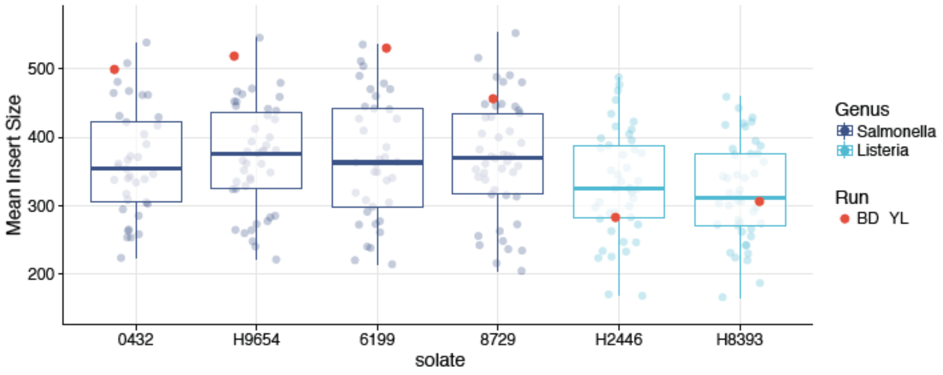

Figure 10: Mean insert size

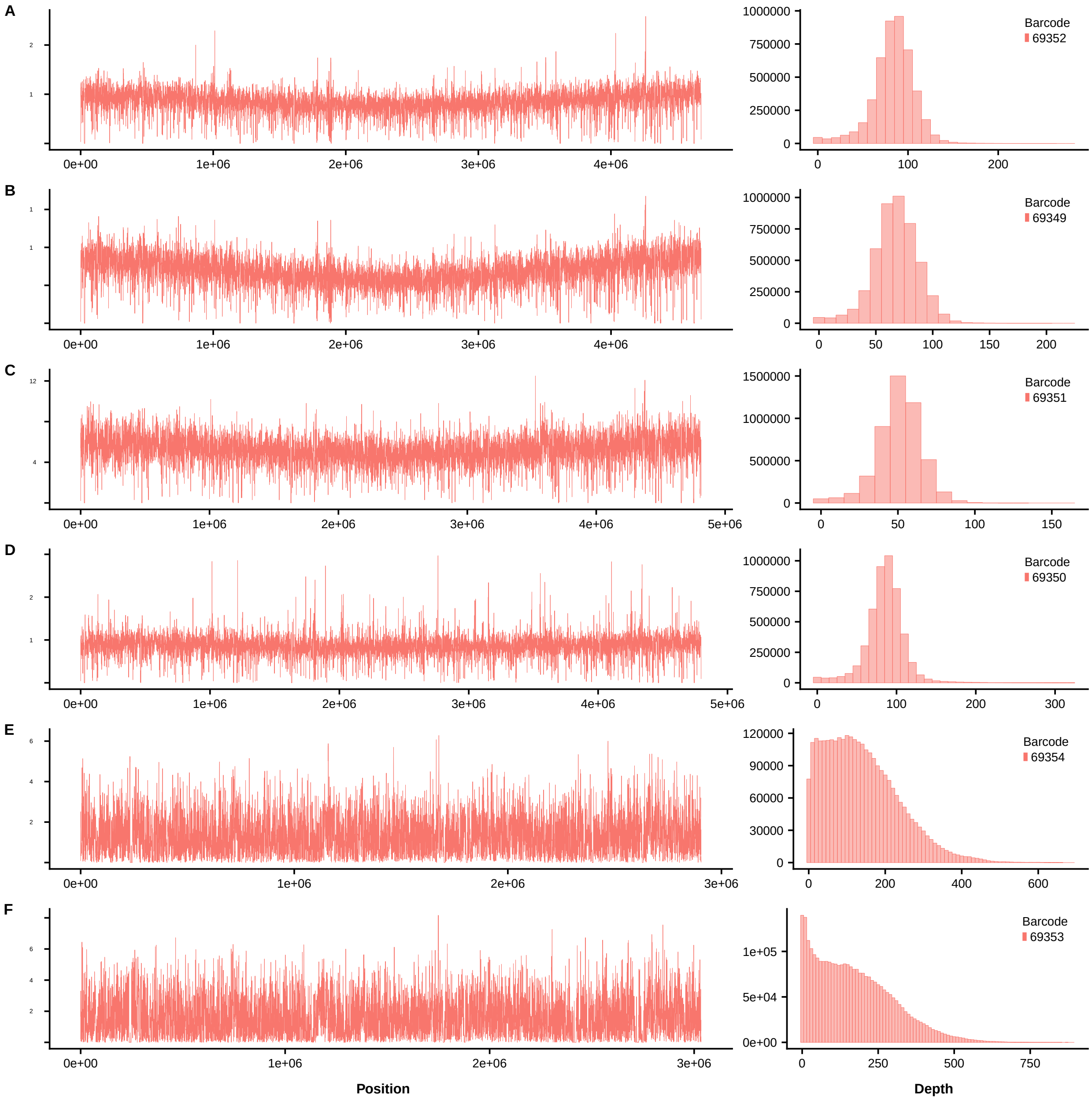

Figure 11: Coverage Plots. A. SAP18-0432 B. SAP18-H9654 C. SAP18-6199 D. SAP18-8729 E. LMP18-H2446 F. LMP18-H8393. Due to the possibility of replicates, the individual coverage datasets are labeled using unique Barcode IDs (see Appendix).

2.4 Assembly Metrics

The following assembly metrics were evaluated (**Figs. 12-15**) -

12. NG50 - The NG50 assembly quality metric. The contig length such that using equal or longer length contigs produces x% of the length of the reference genome, allowing for comparisons between different genomes. Larger NG50 values generally correlate with a higher quality assembly.

13. Contigs - The total number of contigs in the assembly. Fewer contigs generally correlate with a higher quality assembly.

14. Total Length Delta - the difference between the lengths of the assembly and the reference. A perfect assembly would produce a delta of zero. Positive values indicate possible contamination.

15. Unaligned Length - the total length of any assembled contigs which could not be aligned to the reference genome. The presence of unalignable contigs could indicate an unexpected genomic element (plasmid, phage) or contamination.

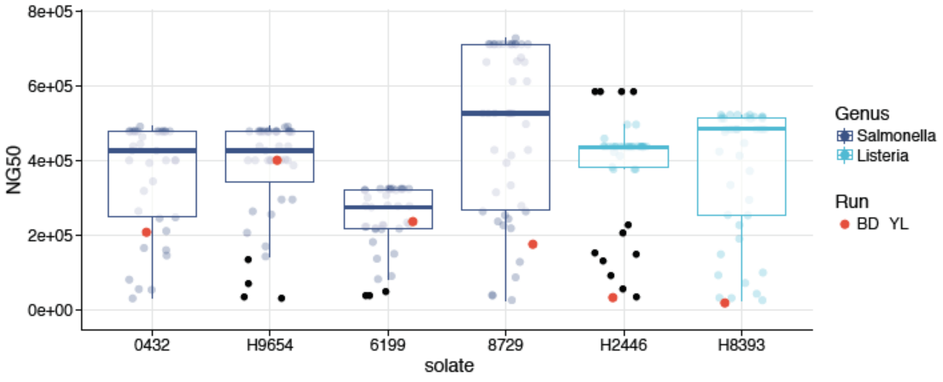

Figure 12: NG50

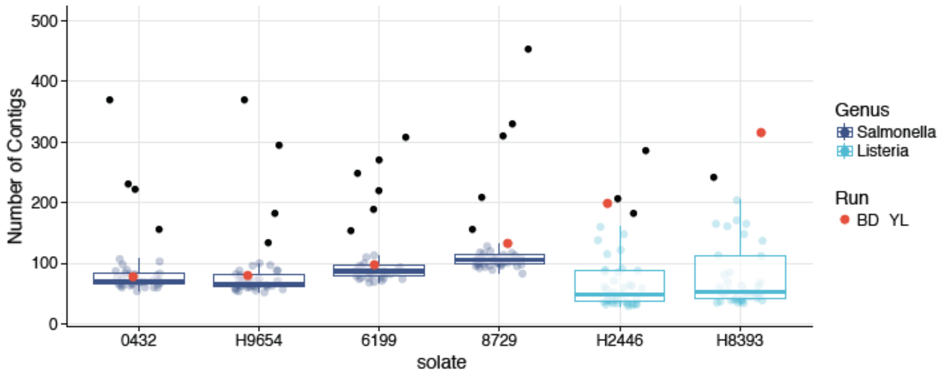

Figure 13: Contigs

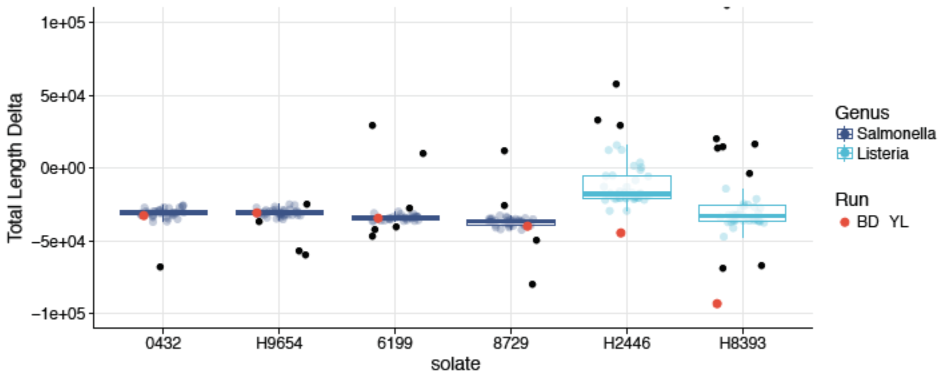

Figure 14: Total Length Delta

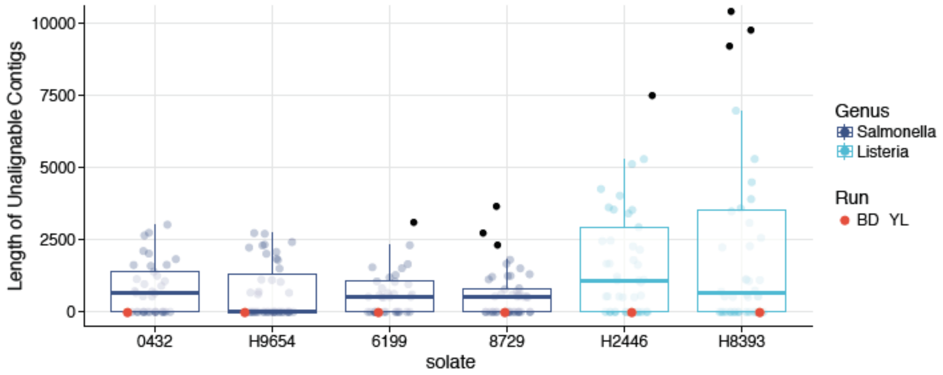

Figure 15: Unaligned Length

3 Conclusions

The assembly quality is worse than expected for 4 of the 6 isolates (Figs. 12 and 13). The Listeria isolates show extreme variance in coverage depth (Figs. 8 and 11), resulting in very poor assembly quality.

Of the 6 PT isolates submitted as part of this exercise, 6 (100%) were determined to be correctly annotated and to have met the minimum thresholds for read depth (20X) and mean read quality (Q27).

Figures 1-15 illustrate the performance of your PT sequencing run(s) based on an evaluation of metrics within the context of the entire distribution of all participants in the 2018 PulseNet-GT harmonized PT exercise. Pay particular attention to any figures in which values for your run fall outside of the 25% - 75% interquartile range (the boundaries of the box in the plot).

A Appendix

	SampleName	Barcode	IsolateID	CFSANID	Lab	Sequencer	Machine	FlowCell	SequencedBy	SeqLength	Reads
1	LMP18-H2446-	SEQ000069354	LMP18-H2446	CFSAN074383		Illumina MiSeq sequence		000000000-BDTYL		35-251	2324708
2	LMP18-H8393-	SEQ000069353	LMP18-H8393	CFSAN074382		Illumina MiSeq sequence		000000000-BDTYL		35-251	2773800
3	SAP18-0432-	SEQ000069352	SAP18-0432	CFSAN074386		Illumina MiSeq sequence		000000000-BDTYL		35-251	1773420
4	SAP18-6199-	SEQ000069351	SAP18-6199	CFSAN074387		Illumina MiSeq sequence		000000000-BDTYL		35-251	1133060
5	SAP18-8729-	SEQ000069350	SAP18-8729	CFSAN074384		Illumina MiSeq sequence		000000000-BDTYL		35-251	2087700
6	SAP18-H9654-	SEQ000069349	SAP18-H9654	CFSAN074385		Illumina MiSeq sequence		000000000-BDTYL		35-251	1423320

Table 4: Run Metadata.

	SampleName	MeanR1	MeanR2	PercMapped	MeanDepth	SNPs	MeanInsert	GenomeFraction	CovLT10	NG50	Contigs	LengthDelta	UnalignedLength
1	LMP18-H2446-	37.20	36.60	99.33	97.09	0	282.80	98.24	138913.00	33574	198	-43927	0
2	LMP18-H8393-	37.00	36.30	99.48	120.69	0	305.26	96.49	221835.00	19439	315	-93313	0
3	SAP18-0432-	35.80	34.00	99.02	70.17	0	499.59	99.09	64229.00	206776	78	-32223	0
4	SAP18-6199-	35.70	33.30	98.81	43.04	1	530.50	99.04	76603.00	238715	97	-34252	0
5	SAP18-8729-	35.90	33.60	98.59	71.66	0	456.42	98.85	85443.00	177181	132	-39517	0
6	SAP18-H9654-	35.80	33.80	98.99	56.75	0	518.08	99.12	65008.00	401093	80	-30348	0

Table 5: Run Metadata Continued.
